# A Unique Case of Membranous Lupus Nephritis Identified After Pronase Digestion

**DOI:** 10.7759/cureus.29083

**Published:** 2022-09-12

**Authors:** Lakshmi Kannan

**Affiliations:** 1 Nephrology, Pikeville Medical Center, Pikeville, USA

**Keywords:** kidney biopsy, nephrotic syndrome, lupus nephritis, pronase, membranous nephropathy

## Abstract

Membranous nephropathy (MN) is a common etiology of nephrotic syndrome (NS) in Caucasian adults. With treatment strategies heavily dependent on differentiating between primary versus secondary MN, tissue diagnosis remains paramount in the setting of indeterminant serological studies and remains the gold standard. Direct immunofluorescence on frozen sections remains standard practice, though with inadequate kidney tissue, antigen retrieval with proteases on formalin-fixed paraffin-embedded tissue can be a viable alternative for direct immunofluorescence. We report a patient who presented with nephrotic syndrome, indeterminant serological workup including primary antigen phospholipase-2 receptor antibody (PLA_2_R). Histology revealed a membranous pattern of injury with a negative standard panel of immunocomplex deposits on direct immunofluorescence. Upon re-examination of paraffin-embedded tissue via protease processing, Immunofluorescence unmasked membranous lupus nephritis. This case highlights the possibility of negative direct immunofluorescence on viable frozen tissue which is unmasked after protease treatment on formalin-fixed paraffin-embedded tissue sample revealing immunocomplex deposits.

## Introduction

Routine tissue processing of kidney biopsy includes sectioning for light microscopy, immunofluorescence (IF), and electron microscopy. IF staining for immunoglobulins and complement components has traditionally been performed on frozen, unfixed cortical tissue samples [1]. When a tissue sample is inadequate for varied reasons, salvage techniques using formalin-fixed, paraffin-embedded tissue (FFPE) have shown to be sensitive and specific [2]. This salvage technique involves the addition of pronase enzyme to unmask antigenic sites, which are crosslinked during fixation, and required for the diagnosis of immune complex-type glomerulopathy [3].

## Case presentation

A 45-year-old Caucasian female with a medical history significant for Type II diabetes mellitus and acquired solitary kidney due to donation in 1999 presented with primary complaints of progressive shortness of breath and associated right lower extremity edema. Pertinent negatives include no history of hypercoagulability, miscarriages, or a family history of autoimmune diseases. In the emergency room, vitals revealed hypertension with a blood pressure of 158/92 mmHg and hypoxia with oxygen saturation of 90% on room air. On physical examination her lung fields were clear but she had right lower extremity 3+ pitting edema up to the level of mid-thigh.

Doppler ultrasound of the right lower extremity was positive for a partially occlusive thrombus in the right femoral vein. Renal ultrasound showed a single right kidney. With further investigation with computed tomography angiogram (CT angiogram) for shortness of breath, submissive pulmonary embolism was discovered. Laboratory workup was significant for creatinine of 1.1 mg/dL at her baseline, albumin at 1.5 g/dL, and no other electrolyte derangements. Urinalysis revealed 3+ protein and moderate haematuria red blood cells (RBC) per high-power field. 24-hour urine collection showed 15.8 g of protein. Expanded serum panel revealed antinuclear antibody positive (>1:160), with slightly low C3 (72 mg/dL) and C4 (10 mg/dL). Furthermore, antibodies to double-stranded DNA, histone, and phospholipase receptor 2 (PLA2R) were negative. Acute hepatitis panel and HIV were also negative. Serum and urine protein electrophoresis was negative with hemoglobin A1c of 5.3%.

**Figure 1:**
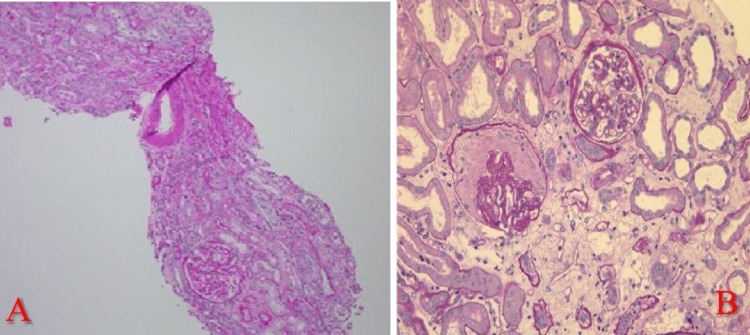
(A) Hematoxylin and Eosin staining of a section of frozen sample showing one normal glomerulus and moderate arteriolar sclerosis. (B) PAS stain shows one glomerulus with global glomerulosclerosis and one normal glomerulus. PAS- Periodic acid Schiff

**Figure 2:**
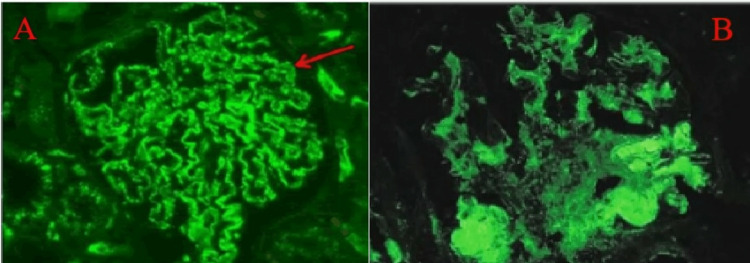
(A) Immunofluorescence staining on an FFPE tissue section after enzymatic digestion with pronase shows fine granular staining for IgG1. (B) staining for C1q. FFPE- Fresh frozen paraffin embedded

**Figure 3:**
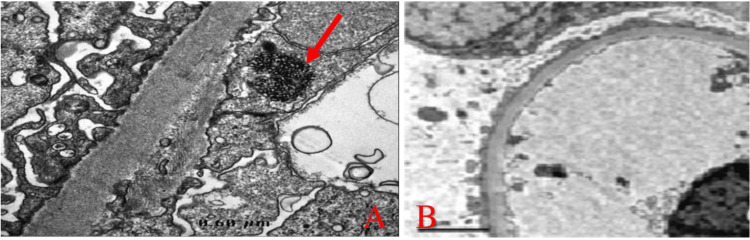
(A) Electron microscopy showing tubulo-reticular inclusion in the endothelial cell cytoplasm. (B) Diffuse foot process effacement.

The patient underwent an ultrasound-guided renal biopsy which contained up to 30 glomeruli by light microscopy; 15% of the glomeruli showed global glomerulosclerosis and moderate arteriosclerosis (Figure 1). Chronic tubulointerstitial fibrosis and atrophy were seen in <10% of the sampled cortex. Direct immunofluorescence (DIF) staining was negative for IgG, IgM, IgA, C1q, C3, kappa, and lambda chains or fibrin. IF on FFPE tissue after pronase digestion showed 3+ fine granular capillary loop IgG1 staining, 1+ IgG2, trace staining with IgG3 and IgG4, 2+ IgM, IgA, C1q, and C3 staining (Figure 2). Negative staining for kappa or lambda light chains and PLA2R. Electron microscopy revealed diffuse foot process effacement, with reticular aggregates seen in the endothelial cell cytoplasm (Figure 3). 

Given the patient's demographics, serology, and the characteristic findings on renal biopsy ‘full house’ staining, she was diagnosed with secondary membranous nephropathy due to lupus nephritis.

Her immunotherapy regimen included prednisone at 1mg/kg for two months, with tapering over five months, and mycophenolate mofetil 1 g twice daily, with a reduction of mycophenolate dose to 750 mg twice daily after three months. On follow-up post-therapy, her urine protein fell to 468 mg/24-hour urine collection with stable serum creatinine between 1.1 and 1.3 mg/dl. Her edema resolved with blood pressures ranging from 125/68 to 145/ 85. She remains in complete remission nine months after initiation of therapy. 

## Discussion

Membranous lupus nephritis or class V lupus nephritis is the most common secondary cause of membranous nephropathy. Some histopathological findings on kidney biopsy characteristic of membranous lupus nephritis include 'full house' staining on biopsy- IgA, IgG, IgM, C1q, and C3, extraglomerular immune deposits in the form of tubular basement membrane staining and vascular deposits, subendothelial electron-dense deposits, and presence of tubuloreticular inclusions [4].

There is an inherent difficulty in distinguishing primary and secondary membranous nephropathy based primarily on light microscopy. Invariably, in order to distinguish the two entities immunofluorescence staining and electron microscopy are required. Direct immunofluorescence on frozen tissue remains the most widely used and gold standard technique, as it is simpler, faster, and more sensitive. However, in the setting of inappropriate frozen tissue samples lacking glomeruli or lacking the required specialized instruments, formalin-fixed, paraffin-embedded tissue sections can be used for IF after a process of antigen retrieval. There are currently two methods of antigen retrieval with varying success rates; Heat-induced antigen revival (HIAT) and enzymatic retrieval such as with pronase [5]. Pronase retrieval was originally described by Fogazzi et al. [6] and has been in practice in the US since 2006.

In our patient, although the suspicion of membranous lupus nephritis was high, direct immunofluorescence was initially negative on a viable tissue sample with adequate glomeruli, likely typical of pauci-immune glomerulonephritis, which has also been compared with a control sample at the laboratory. With the incongruous results, we decided upon additional testing on the FFPE tissue through a pronase unmasking process. This was done assuming 1) formalin fixation preserves tissue morphology by cross-linking proteins; 2) pronase digestion unmasks antigenic sites [7].

Certain glomerulopathies, membranous-like glomerulopathy with masked IgG kappa deposits, and membranoproliferative glomerulonephritis (MPGN) with masked monotypic Ig deposits require immunofluorescence on paraffin fixated tissue for diagnosis [4]. However, negative staining for kappa and lambda light chains on FFPE tissue in this patient ruled out dysproteinaemia-associated renal disease.

Direct IF is the gold standard immunohistochemical technique in renal pathology, and FFPE is not necessary in most cases. The indications for FFPE are 1) frozen tissue lacks glomeruli; 2) suspected light chain nephropathy; 3) MPGN with negative staining for Igs; 4) C3 glomerulonephropathy associated with monoclonal gammopathy; 5) membranous nephropathy with negative Ig staining; 6) fibrillary glomerulonephritis with monotypic IgG deposits by IF [8].

An important flaw in paraffin immunofluorescence is that due to fixation, the glomerular capillaries will often have residual serum, which is typically not present in the routine immunofluorescence section, that could stain non-specifically positive for some antibodies and differences in granular staining [7].

## Conclusions

Even though it is still mysterious why the glomerular deposits stained on FFPE and not on DIF in this case of class V lupus nephritis, FFPE remains a valuable, salvage, and unmasking technique for renal pathology when the index of suspicion is high in order to prevent misdiagnosis due to masked immune complex deposits. 
